# Aquaporin membrane channels in the hepatobiliary tract: a model of complexity and clinical implications in health and disease

**DOI:** 10.1007/s11739-026-04332-y

**Published:** 2026-04-02

**Authors:** Piero Portincasa, Mohamad Khalil, Giuseppe Calamita

**Affiliations:** 1 Clinica Medica “A. Murri”, Department of Precision and Regenerative Medicine and Ionian Area (DiMePre-J), University of Bari “Aldo Moro” Medical School, Bari, Italy; 2 Department of Biosciences, Biotechnologies and Environment, University of Bari “Aldo Moro”, Bari, Italy

**Keywords:** Aquaporins, Hebatobiliary system, Bile secretion, Cholestasis

## Abstract

Aquaporins (AQPs) are transmembrane channel proteins that allow water and certain small solutes to pass through. In the liver, bile ducts, and gallbladder, AQPs are crucial in the pathways of hepatobiliary secretion and reabsorption. AQP8 and AQP9 are essential for facilitating the osmotic movement of water in hepatic bile, which is 95% water. In the biliary tract, AQP1 and AQP4 are involved in the rearrangement of bile composition by mechanisms of reabsorption/secretion of water. In the gallbladder, AQP1 and AQP8 are also involved in trans-epithelial bidirectional water flow with the ultimate goal of bile concentration. Pathophysiologically, AQPs have been indicated as players in several hepatobiliary disorders, including cholestatic diseases and cholesterol cholelithiasis. Research is ongoing to fully understand AQP function and how its expression is regulated, with scientists discovering powerful and specific compounds that can either boost or block these membrane channels, potentially leading to significant pharmaceutical advancements. This review summarizes the contribution of AQPs in physiological and pathophysiological stages related to hepatobiliary function.

**Supplementary Information:**

The online version contains supplementary material available at 10.1007/s11739-026-04332-y.

## Introduction

Bile is a yellowish-green digestive fluid produced by the liver, temporarily stored in the gallbladder before entering the duodenum and the remaining gut. It mainly consists of water, lipids, and waste substances such as bilirubin. Three main biliary lipid species are cholesterol, phospholipids, and bile acids (BAs) [1]. Additional solutes include bilirubinate pigments, small quantities of proteins, and inorganic salts [2]. Minor concentrations of proteins and metabolites of endogenous compounds like hormones, such as those listed in references [3, 4] are present. The major biliary inorganic ions found in the common duct bile are Na^+^, K^+^, Ca^2+^, Mg^2+^, Cl^−^, and HCO_3_^−^, with concentrations like those in plasma. Following liver production and secretion, the diluted bile enters the gallbladder, where it becomes concentrated during times of fasting. After a meal high in fat and a neurohormonal stimulus leading to gallbladder contraction, the bile that is released enters the duodenum and flows across the intestinal tract [5, 6]. Bile plays a key role during digestion through enhanced emulsification and absorption of dietary lipids, including triglycerides and cholesterol, as well as fat-soluble vitamins such as A, D, E, and K. Furthermore, bile is the only route which allow the removal of cholesterol from the body with BAs and phospholipids (as > 95% lecithins) functioning as cholesterol carriers to keep cholesterol solubilized in the watery part of bile. The aggregation of biliary lipid species begins on the canaliculus side of hepatocytes, where simple and mixed micelles (enriched in BAs), and unilamellar and multilamellar vesicles form, depending on the concentration of cholesterol and BAs [7].

**Fig. 1:**
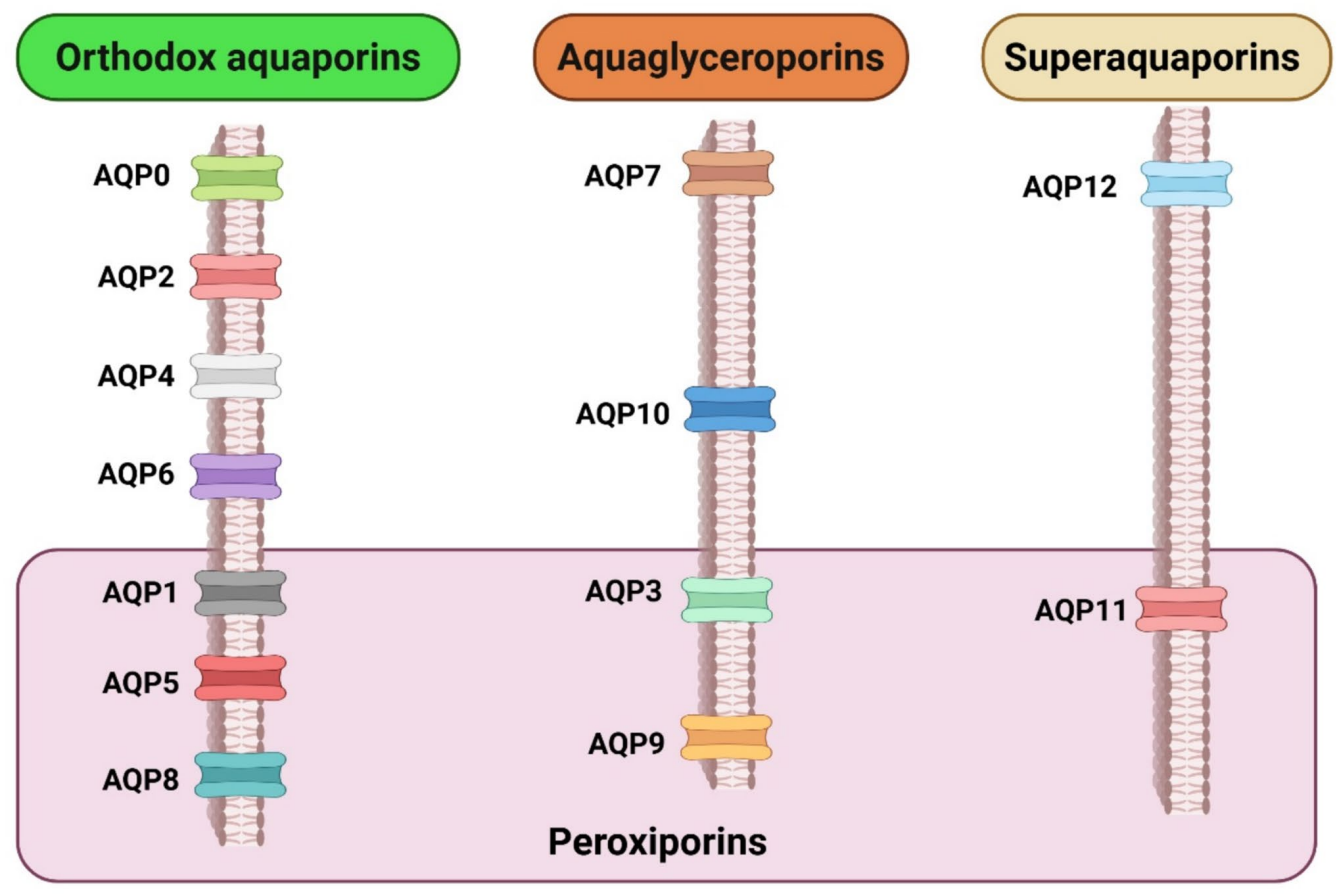
Schematic representation of the thirteen human aquaporin membrane channels (AQPs), categorized into four functional groups based on their molecular selectivity: Orthodox Aquaporins (AQP0, AQP1, AQP2, AQP4, AQP5, AQP6, AQP8), Aquaglyceroporins (AQP3, AQP7, AQP9, AQP10), Superaquaporins (AQP11, AQP12), and Peroxiporins (AQP1, AQP3, AQP5, AQP8, AQP9, AQP11). Occasionally, a fifth group of AQPs characterized by expressing ammonium permeability, the so-called Aquaammoniaporins (AQP1, AQP3, AQP6, AQP7, AQP8 and AQP9) has been described (not shown in the figure). Water, glycerol, hydrogen peroxide and ammonia are not the only molecules towards which AQPs are permeable (see the Introduction). Created in https://BioRender.com

Aquaporins (AQPs) are membrane proteins that facilitate single-file diffusion of water and certain small neutral solutes through pores in each monomer of their tetrameric structure [8]. Humans possess thirteen distinct AQPs (AQP0–12) roughly subdivided into three groups based on their molecular selectivity and sequence similarity: (1) the orthodox aquaporins (AQP0, AQP1, AQP2, AQP4, AQP6, and AQP8), (2) the aquaglyceroporins (AQP3, AQP7, AQP9, and AQP10), and (3) the superaquaporins (unorthodox aquaporins, AQP11 and AQP12) [9, 10]. However, this classification does not adequately delineate the molecular selectivity of AQPs, so depending on their additional permeability to hydrogen peroxide and ammonia, the names as peroxiporins (AQP1, AQP3, AQP5, AQP8, AQP9, and AQP11) or aquaammoniaporins (AQP1, AQP3, AQP6, AQP7, AQP8, and AQP9) are also used. AQPs are also involved in the transmembrane movement of substances such as urea (AQP3, AQP6, AQP7, AQP9, AQP10), nitric oxide (AQP1, AQP4), and gases including oxygen (AQP1) and carbon dioxide (AQP0, AQP1, AQP4, AQP5, AQP6, AQP9) [11]. In addition, certain AQPs have been reported to facilitate the diffusion of inorganic ions (AQP0, AQP1, AQP6), silicon (AQP3, AQP7, AQP9, AQP10), weak monoacid anions such as lactate (AQP9), and antimonite and arsenite (AQP7, AQP9) [12, 13] (Fig. 1).

Due to the pleiotropic role of AQPs in the biliary scenarios, this review examines the physiological implications of different AQPs in bile secretion and reabsorption processes. Natural and synthetic compounds are studied for their effects on the increased or decreased expression and function of AQPs. This approach helps clarify the pathophysiological regulation of biliary water transport. Research in this area is still being conducted with the goal of treating biliary diseases using agonists, antagonists, gene therapies, and other methods. While previous preclinical research has yielded encouraging findings, consideration must be given to factors such as disease mechanisms, desired therapeutic outcome, safety, and effectiveness. Based on these assumptions, the following is an updated and thorough examination of critical physio-pathological aspects of AQP function in health and disease, along with insights into potential and innovative methods to modulate the molecular permeability of AQPs as additional therapeutic targets.

## Literature search strategy and selection criteria

This comprehensive review was informed by a structured literature search conducted in PubMed/MEDLINE, Web of Science, and Scopus databases, covering publications available up to December 2025, with no lower limit on publication year to include seminal studies. Search terms included combinations of *aquaporin*, *aquaglyceroporin*, *AQP*, and specific isoforms (AQP1, AQP3, AQP4, AQP5, AQP8, AQP9, AQP11) together with terms related to the hepatobiliary system (*liver*, *hepatocyte*, *cholangiocyte*, *bile duct*, *bile formation*, *gallbladder*) and associated physiological and pathological conditions (*cholestasis*, *bile secretion*, *gallstone disease*, *MASLD/NAFLD*, *liver cancer*). Original research articles and relevant review papers published in English were included if they addressed AQP expression, regulation, molecular mechanisms, or functional roles in hepatobiliary physiology or disease. Reference lists of key articles were manually screened to identify additional seminal and recent studies. The final selection integrates foundational discoveries with recent advances to provide a comprehensive and analytically structured overview of the field.

The selection process is summarized in a PRISMA-style flow diagram (Supplementary Fig. 1).

## The aquaporin family of membrane channels

The existence of channel proteins facilitating the movement of water across biological membranes had been predicted well in advance [14–21] before their molecular identity and experimental functional evidence were discovered and demonstrated by Peter Agre and his collaborators in 1992 [22]. The seminal discovery of AQPs earned Peter Agre the Nobel Prize in Chemistry in 2003 [8]. AQPs play a vital role in maintaining body homeostasis, with their distribution, expression, and function regulated under both normal and pathological conditions through post-translational modifications, such as phosphorylation, ubiquitination, glycosylation, subcellular localization, degradation, and protein interactions [23]. AQPs are synthesized and inserted into the endoplasmic reticulum membrane and are ultimately targeted to the target membrane. Some AQPs are initially present in intracellular vesicles, but later relocate to the plasma membrane. AQP2 and AQP8 move from intracellular vesicles to the plasma membrane in renal collecting ducts and rat hepatocytes in response to vasopressin and cAMP, respectively [24, 25]. AQP trafficking is a highly dynamic process, initially targeting the plasma membrane for removal from the membrane, and its subsequent degradation or recycling in the endosome. Notably, the incorrect placement of AQPs may result in certain human congenital disorders, as in the case of nephrogenic diabetes insipidus (NDI) resulting from AQP2 mislocalization [26]. The predominantly expressed AQP isoform in the brain, AQP4, localizes by means of two C-terminus motifs: a tyrosine motif (Yxx8; 8, V/L/I/F) and a dileucine-like motif [27, 28]. One of the key regulatory mechanisms involved in both the control of AQPs and their movement is phosphorylation [23]. The precise translocation of AQP2 to the plasma membrane from intracellular vesicles following vasopressin treatment requires the phosphorylation of the C-terminus Ser256 [29]. Phosphorylation of Ser261 has been identified in AQP2 located within vesicles, requiring subsequent dephosphorylation for relocation to the plasma membrane [30]. In human neutrophils, AQP9 insertion in the plasma membrane depends on phosphorylation at Ser11, which appeared to be regulated through a Rac1-dependent pathway [31]. Conversely, the recycling of AQPs is a significant cellular process, and research has demonstrated that AQPs are ubiquitinated to regulate their degradation [32]. AQP2 is targeted for ubiquitination at Lys270, leading to its internalization in kidney collecting duct cells [32]. Regulation of some mammalian AQPs through pH-dependent gating has been proposed by some authors not to involve the movement of water or neutral solutes but rather to involve a presumed permeability to ions through a tetrameric central pore [33–35]. However, the topic remains rather debated.

Aquaporin dysregulation has been linked to a variety of diseases [36]. Listed among these conditions are metabolic syndrome [37], cardiovascular diseases [38], renal concentration disorders [39], obesity [40], diabetes [41], liver steatosis [42], and gallstones [43]. AQPs have also been linked to cancer, inflammation, and a variety of diseases [44–48]. They are also expressed in different cell types within the hepatobiliary system [49]. Hepatocytes, express AQP8, AQP9, and AQP11. AQP8 exhibits multiple subcellular localizations and is involved in the processes of bile water secretion, ammonia detoxification, redox balance, and cholesterol biosynthesis. AQP9 is localized to the basolateral plasma membrane, where it plays a role in the uptake of glycerol, urea, and other solutes [50, 51]. The epithelial cells lining the bile ducts, known as cholangiocytes, are responsible for secreting bile through the ducts. AQP1 is expressed in cholangiocytes and plays a role in water flow during bile formation. The intestinal hormone secretin manages water secretion, which is facilitated by AQP1, under the control of cAMP signaling [52]. The gallbladder stores and concentrates bile and this function depends on gallbladder epithelial cells which express AQP1 and AQP8 facilitating bidirectional water flow across the gallbladder wall [53].

Observations of dysregulated AQPs have been reported in several hepatobiliary diseases.

## Hepatobiliary AQPs in bile formation and flow

Bile is made of ~ 95% water [7, 54], and in healthy conditions, adult humans secrete about 0.8–1.0 L of hepatic bile daily at a rate of 30–40 mL per hour [7]. Bile production is approximately six times greater in rats, a species which does not possess a gallbladder [1]. Formation of bile fluid starts at the canalicular (apical) membrane of hepatocytes as an osmotic process involving solutes and water. BAs and other biliary substances are actively secreted into bile canaliculi, which produces the osmotic pressure that drives parallel water secretion [1]. In the absence of BAs or at low BA outputs, BA-independent bile flow also occurs, as canalicular bile flow is also found. The extra bile flow component is attributed to the active secretion of osmotically active inorganic electrolytes and organic anions. Total bile production arises from continuous ductal secretion and the overall flow of bile into canaliculi, consistent with the linear relationship observed in both total bile flow and total canalicular bile flow [1]. Total bile formation results from constant ductal secretion and total canalicular bile flow, consistent with the linear relation existing in both total bile flow and total canalicular bile flow [1].

The process of enterohepatic circulation of bile with its solutes is a complex one and is primarily governed by the BA pool. The “primary” BAs are synthesized in the liver from cholesterol and consist of the trihydroxy cholic acid (CA) and the dihydroxy chenodeoxycholic acid (CDCA). This step is followed by the conjugation of BAs with taurine and glycine to increase their solubility. Following gallbladder storage and contraction, the BAs flow across the gut. Approximately 5% of primary BAs evade the active ileal absorption and reach the colon, where the resident microbiota begins the process of deconjugating BAs from taurine and glycine, as well as dehydrogenation, dehydroxylation, and epimerization, ultimately producing the “secondary” BAs consisting of dihydroxy deoxycholic acid (DCA) and monohydroxy lithocholic acid (LCA). The 7α-dehydrogenation of CDCA leads to the formation of dihydroxy 7α-oxo (keto)-LCA, which is then metabolized into the “tertiary” 7β-epimer, dihydroxy ursodeoxycholic acid (UDCA), in the colon, and back into CDCA in the liver. LCA, 7-oxo (keto)-LCA, and UDCA are primarily excreted through feces, whereas approximately 50% of DCA is passively reabsorbed from the colon into the portal tract [55] via ionic diffusion more than nonionic diffusion [56]. In total, the BA pool in each cycle undergoes re-conjugation with taurine and glycine and new secretion into bile. Fecal loss is less than 5% in each cycle. For instance, if a CA or CDCA pool weighing 1 g undergoes 6 cycles daily, the daily loss amounts to 5% per cycle multiplied by 6 cycles equals 30%, and 300 mg must be re-synthesized in the liver [57].

**Fig. 2:**
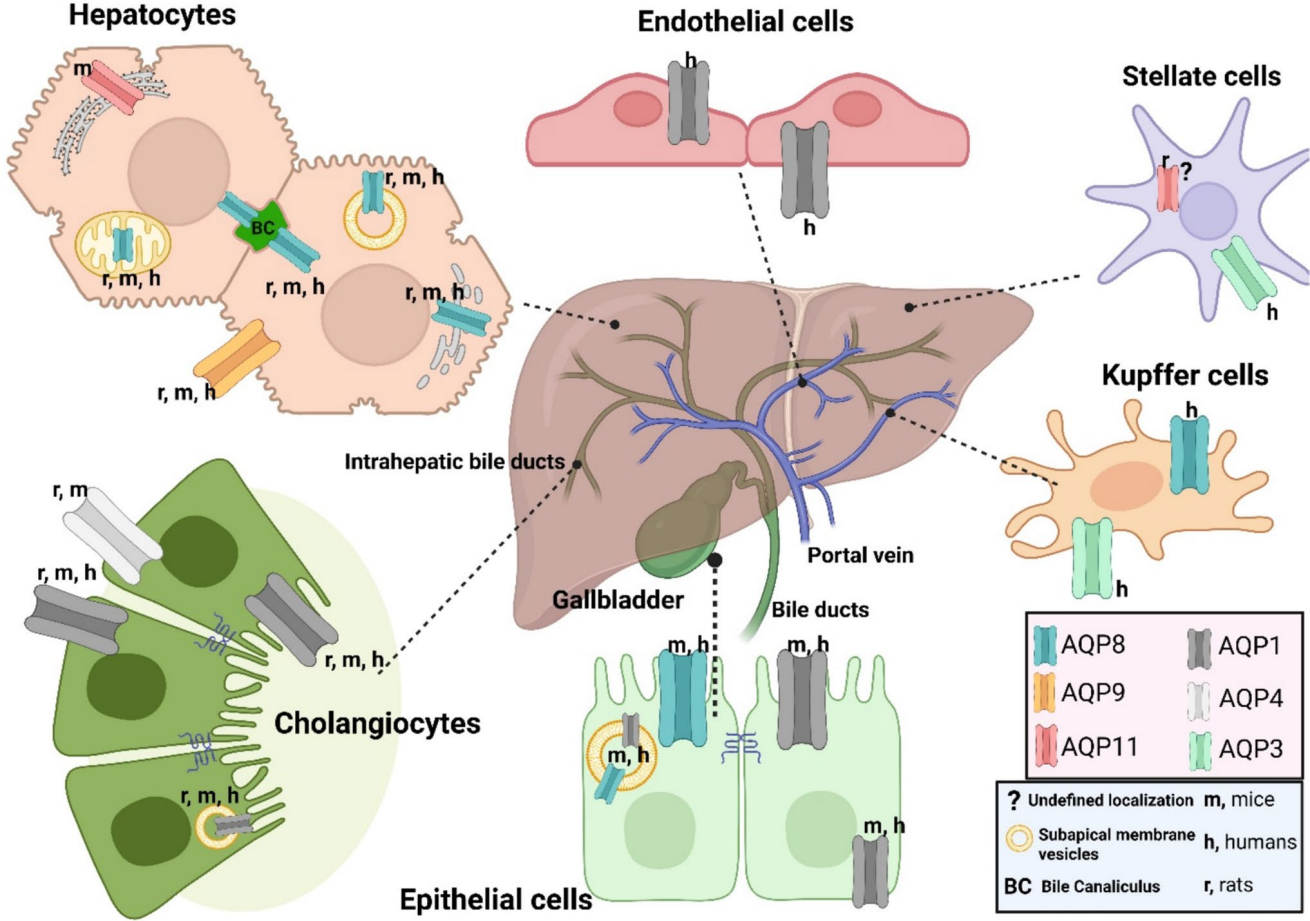
Schematic representation of AQP distribution and subcellular localization in the hepatobiliary tract across humans and rodents. Cells involved are hepatocytes, cholangiocytes, endothelial cells, hepatic stellate cells, Kupffer cells, and epithelial cells of the bile ducts and gallbladder. Created in https://BioRender.com

Several epithelial cells lining the mammalian hepatobiliary tree express multiple AQPs with unique subcellular locations and functions (Fig. 2). Like other body regions, the blood vessels of the hepatobiliary system also express AQP1 [58]. Supplementary Fig. 2 explains the cell type-specific role of AQPs in bile formation and flow along the hepatobiliary axis. In hepatocytes, AQPs at the canalicular membrane facilitate osmotic water movement into the bile canaliculus in response to solute secretion, with cAMP/PKA-dependent trafficking increasing membrane water permeability. In cholangiocytes, secretin-induced cAMP signaling promotes coordinated activation of CFTR and AE2, generating osmotic gradients that drive AQP-mediated ductal fluid secretion and contribute to bile salt-independent bile flow. In gallbladder epithelial cells, AQPs at apical and basolateral membranes enable rapid water reabsorption, allowing bile concentration during storage. Together, the figure illustrates how regulated AQP expression and trafficking govern bile formation, ductal modification, and final bile composition.

## Relevance of AQPs in the hepatobiliary tract

### Liver

Hepatocytes from liver, human and rodent express high levels of AQP8 and AQP9. In addition to mouse hepatocytes, AQP11 is also expressed by Huh-7 cells, a line of immortalized human hepatocytes [59], as reported in Ref. [60]. Human liver cells also express AQP3. The function of AQPs in human liver cells remains unclear, but it is plausible that their redundancy is justified by their distinct molecular preferences and subcellular localization [50]. While AQP8, AQP9, and AQP11 play crucial roles, the function of AQP3 in hepatocytes [61] is uncertain with respect to any ultimate physiological significance. In hepatocytes, AQP8 has multiple subcellular localizations, including the canalicular membrane, subapical vesicles, and various organelles like mitochondria and the smooth endoplasmic reticulum [62, 63]. Hepatocyte AQP8 is known to perform several functions, such as facilitating the secretion of bile water into canaliculi [43, 64, 65], maintaining cytoplasm osmolarity during glycogen synthesis or breakdown [63], facilitating the movement of ammonia in mitochondrial ammonium detoxification and urea production [66–69], and mediating the efflux of hydrogen peroxide from mitochondria during oxidative stress [70, 71]. Research using human Huh-7 cells and rat liver cells indicated that mitochondrial AQP8 is involved in the production of cholesterol in liver cells, which is regulated by the sterol regulatory element-binding protein (SREBP) [72–74]. A recent study with obese mice found that overexpressing AQP8 in the liver led to the activation of the Farnesoid X receptor (FXR), resulting in the inhibition of genes involved in lipogenesis and a further decrease in intrahepatic triacylglycerol (TAG) overaccumulation [75]. AQP8 is likely involved in the differential regulation of metabolic signaling by α1- and β-adrenoceptors (ARs) due to its peroxiporin property, leading to the induction of Ca^2+^ ion mobilization. Hydrogen peroxide blocked the β-AR-mediated activation of the glycogenolytic, gluconeogenic, and ureagenic responses caused by α1-AR, the NOX2–H_2_O_2_–AQP8–Ca^2+^ signaling pathway, an observation which led to the hypothesis that the signaling related to H_2_O_2_ passing through AQP8 constitutes an additional pathway acting downstream of α1-AR in hepatocytes. Hydrogen peroxide’s inhibitory effect on β-AR signaling could also account for the negative interaction between the two signaling pathways [76]. Substantial research has been conducted to clarify the function of AQP8 in the secretion of canalicular bile [50, 64]. Agonists of choleretic properties, such as dibutyryl cyclic adenosine monophosphate (cAMP) and glucagon, were found to initiate the movement of subapical vesicles containing AQP8 to the canalicular plasma membrane via a pathway associated with phosphatidylinositol-3-kinase and microtubules [77]. Translocation of AQP8 into the apical membrane of hepatocytes was associated with an increase in the water permeability of the canalicular plasma membrane and the osmotic movement of water into the bile canaliculus [25, 43, 65, 78] (Fig. 2). Redistribution from the cytoplasmic compartment to the apical membrane of hepatocytes, induced by cyclic AMP, also occurs for molecular carriers involved in canalicular bile secretion, such as the isoform 2 of the Cl^−^/HCO_3_^−^ exchanger (AE2) and multidrug resistance-associated protein 2 (MRP2). The implication of AQP8 in hepatic bile formation was argued in a study with hepatocytes from *Aqp8*^−/−^ knockout mice where the canalicular osmotic water permeability was reported to be of a similar extent to that of *Aqp8*^+/+^ wild-type mice [79]. This discrepancy may be accounted for by the redundancy of AQP transporters in liver cells and by the fact that the osmotic water permeability of the hepatocyte canalicular membrane is not entirely dependent on AQP8. A study of rat primary hepatocytes found that the water permeability of the canalicular membrane is not entirely dependent on AQP8, as a 60% decrease in AQP8 levels in the apical membrane resulted in a 15% reduction in overall canalicular osmotic permeability [79]. The concept that water flow into the canaliculus is dependent solely on the rate of carrier-mediated transport has recently been questioned by changes in hepatic bile composition observed in Claudin-2 knockout mice and following examination of the cholestatic effect of estradiol 17β-d-glucuronide [80]. A reduction in paracellular or transcellular canalicular water flow, possibly mediated by AQP8, did not reveal any considerable impact on bile acid secretion. A recent study involving *Aqp8*^−/−^ knockout mice found that they exhibited decreased canalicular bile formation, characterized by the secretion of concentrated bile with a lower flow rate and higher levels of bile lipids compared to their *Aqp8*^+/+^ littermates [43].

AQP9 is a widely selective aquaglyceroporin that besides water enables the movement of a large range of neutral solutes, such as glycerol and other polyols, H_2_O_2_, urea, carbamides, nucleosides, monocarboxylates, purines, pyrimidines, and metalloid arsenic [81, 82]. This aquaglyceroporin has been assigned multiple functions [51]. AQP9 is found at the sinusoidal domain of the basolateral plasma membrane in both rodent and human hepatocytes [83]. In rodents, AQP9 is the primary route through which glycerol is absorbed by liver cells from portal blood during fasting [84–86]. Glycerol imported into the body is quickly converted into glycerol-3-phosphate, a key substrate for gluconeogenesis during the initial stages of starvation. AQP9 also plays a role in lipid homeostasis due to the need for glycerol-3-phosphate in the synthesis of triacylglycerol [87]. Hepatocyte AQP9 is also proposed to play a role in rodent bile production and in removing catabolic urea from the body by enabling the entry of water and the exit of urea into and out of the portal blood, respectively [88, 89]. In rodents, insulin negatively regulates AQP9 expression at the transcriptional level [90], which is why AQP9 levels are increased in the liver during insulin resistance [88, 89]. Studies on AQP9-depleted knockout mice indicated the functional relevance of AQP9 in metabolic homeostasis and energy balance, showing that the absence of AQP9 was linked to reduced liver glycerol permeability and higher levels of plasma glycerol and TAGs [89, 91]. In murine models of obesity and in individuals living with obesity and type 2 diabetes, reduced AQP9 levels were observed in hepatocytes along with significant decrease in liver glycerol permeability [92, 93]. The expression of AQP9 in the liver is also affected by leptin [87, 94], a peptide hormone produced in fat cells, the placenta, and, to a lesser extent, the gut. Leptin reflects both energy stores, such as mainly fat, and energy equilibrium, such as weight loss or maintenance, and binds to leptin receptors on the surface of neurons in the hypothalamus to modulate food consumption and energy equilibrium [95]. The modulation of AQP9 by both insulin and leptin seems to vary between humans and rodents, as indicated in Ref. [61], and this disparity warrants further research. Liver AQP9 expression is reported to exhibit sex-related differences in both rodents and humans, consistent with established differences between the sexes in how glycerol is handled for metabolic purposes [94, 96]. AQP3 and AQP7, two other AQPs of metabolic importance in adipose tissue, also exhibit sex-specific differences [94, 97]. The AQP9 gene played a part in the cholesterol-lowering effect of silybin, a plant-derived supplement, when tested in rat liver cancer cells, through the regulation of autophagy and lipid droplet formation [98]. AQP9 in the liver has also been found to be highly relevant to the immune system. TLR4 ligands, such as LPS, have been reported to engage AQP9 in the mechanism that leads to the production of inflammatory NO and O_2_^−^ through the involvement of the NF-kB pathway [99] and the NLRP3 inflammasome [100]. A subsequent study found that the inhibition of AQP9 by the specific and potent blocker RG100204 eliminates the LPS-induced increase in NO and O_2_^−^ in FaO cells, a rat hepatoma cell line [101]. Additional research is required to fully grasp the part hepatic AQP9 plays in the immune system as a study has found that *Aqp9*^−/−^ knockout mice have an increase in B and CD4 + T cells through increased cathepsin S and macrophage activation, leading to an immune and inflammatory response [102].

AQP11 in the mouse liver has been proposed to play a role in maintaining rough endoplasmic reticulum balance and liver regrowth, but the precise mechanism by which this unorthodox aquaporin intervenes remains unclear [59, 103]. The recent discovery of AQP11 as a peroxiporin has opened new possibilities for its potential role in maintaining intracellular H_2_O_2_ balance and preventing ER stress [10]. Studies have suggested that AQP11 facilitates the transfer of H_2_O_2_ from mitochondria to the endoplasmic reticulum as part of an interorganellar redox response that is triggered by the downregulation of endoplasmic reticulum flavoenzyme oxidoreductin-1α (ERO1α) [104]. Further studies will therefore be expected to evaluate the precise function of AQP11 in the liver.

### Bile ducts

The epithelial cells lining the biliary tree, known as cholangiocytes, play a crucial role in ductal bile secretion mediated by a cAMP-dependent pathway [105] and activation of the cystic fibrosis transmembrane conductance regulator (CFTR), which results in the extrusion of HCO_3_^−^ into the lumen through apical AE2 and Cl^−^ efflux. HCO_3_^−^ and Cl^−^ are the primary driving forces for the osmotic movement of water through apical AQP1 into the biliary lumen [105, 106]. AQP1 in human and rodent cholangiocytes is responsible for the apical secretion of water during both basal- and hormone-regulated ductal bile formation [58, 107, 108]. AQP1 is also found in subapical membrane vesicles [109], where it is co-expressed with AE2 and CFTR [110], and secretin has been reported to regulate the insertion of these vesicles into the apical membrane of cholangiocytes [61, 109]. This led to the development of a new paradigm for the functional bile secretory unit. The cells lining bile ducts have AQP4 and AQP1 at their basolateral membranes [109, 111], allowing water movement that helps maintain a stable concentration of bile as it forms. This association is also in line with the physical connection between the basolateral membrane of cholangiocytes and the peribiliary vascular plexus surrounding the bile ducts, where bile water originates [50, 112]. The water permeability of cholangiocytes isolated from *Aqp1*^−/−^ knockout mice did not result lower than that of those isolated from the *Aqp1*^+/+^ wild-type mice [113]. It was hypothesized that compensatory upregulation of other mouse cholangiocytes AQPs, such as AQP8, could explain this unexpected observation [114, 115]. Intrahepatic bile ducts secrete and absorb water, as demonstrated by experiments conducted on isolated rodent intrahepatic bile duct units [116]. The osmotic absorption of water is probably initiated by the active uptake of sodium-coupled glucose and BAs via the SGLT1 and ASBT cotransporters, respectively [105]. Hormones like somatostatin, gastrin, and insulin, which reduce intracellular cAMP levels in cholangiocytes, may work by blocking the transport of AQP1, CFTR, and AE2 to the apical membranes of these cells, resulting in decreased bile secretion from the bile ducts [117]. This mechanism may also explain why somatostatin decreases ductal secretion while increasing the net absorption of ductal water. A functional association between CFTR and AQPs has been described in murine Sertoli cells [118, 119].

### Gallbladder

The mammalian gallbladder functions as a dynamic reservoir for diluted hepatic bile, which flows bidirectionally through the cystic duct. Daily fluctuations in gallbladder emptying and refilling are controlled by neurohormonal signals and are crucial for lipid digestion and maintaining metabolic balance, as well as the enterohepatic circulation of bile acids [5–7, 56, 120]. The concentration of stored bile in the gallbladder increases during fasting and is influenced by the movement of water across the gallbladder’s epithelial lining. This step is driven by osmotic gradients produced as a result of active salt absorption and secretion [50, 121]. Human and mice gallbladder epithelial cells express AQP1 and AQP8. AQP1 is located on both the apical and basolateral plasma membranes of the epithelial cells surrounding the neck of the organ [122]. AQP1 is also found in the corpus portion of the gallbladder where immunoreactivity has been observed at the plasma membrane and over subapical vesicles, which can be incorporated into the apical membrane by a microtubule-dependent and cAMP-stimulated mechanism, although the stimulation of this mechanism is still unknown [123]. AQP1 was found to be slightly upregulated by leptin in murine gallbladder [124]. The AQP8 protein has been found at the plasma membrane and, to a lesser degree, in intracellular vesicles of the gallbladder epithelium of various species [58, 62]. A recent study found that the liver X receptor β (LXRβ), an oxysterol-activated transcription factor mainly present in the gallbladder epithelium [53], causes an increase in gallbladder cholangiocytes expression of AQP1 and AQP8, as well as CFTR. A molecular partnership has been reported between CFTR and AQPs in mouse Sertoli cells [119]. A study discovered high water permeability in mouse gallbladder epithelium, with a permeability factor of 0.2 cm/s, enabled by transcellular water transport through AQP1 in wild-type mice [125]. Studies have also found that osmotic water permeability is not dependent on cAMP levels and is unaffected by the size or direction of an osmotic gradient. Similar to bile duct cholangiocytes, a functional equivalent of the gallbladder epithelium, subapical AQP1 was proposed to relocate to the apical membrane to secrete water. Gallbladder AQP8 was speculated to facilitate the absorption of water and, to a lesser degree, to secrete water into the lumen, based on its subcellular pattern of localization [62].

The physiological role of AQP1 and AQP8 in gallbladder function is still a matter of debate. Inconsistencies in reported findings are observed in the literature. Researchers discovered comparable BA levels in gallbladders from both wild-type and AQP1-deficient mice, with no indication that AQP8 took over AQP1’s role [125]. This finding, nonetheless, was inconsistent with earlier studies that reported a time-related link between a reduction in gallbladder concentrating ability and decreased AQP1 or AQP8 levels [123], and the results were derived from leptin-deleted mice that had undergone leptin replacement therapy where the hormone affected the gallbladder volume by influencing the AQP-mediated absorption or secretion of water [126]. Further work is therefore needed to clarify the question more precisely, also taking into account the paracellular pathway since the gallbladder lumen is lined with a leaky epithelium [127].

## AQPs in hepatobiliary diseases

Diseases of the hepatobiliary tree are linked to the disruption of bile transport and impaired bile flow [50, 128]. Experimental models of cholestasis have shown that abnormal AQPs in the hepatobiliary system and impaired bile secretion are present, and research conducted using cellular and mouse models of gallstones indicates a link between changes in the expression and localization of AQP in cholangiocytes and the ability of the gallbladder to concentrate.

### Liver

AQP proteins have a substantial role in the development and progression of hepatocellular carcinoma. AQP3 is overexpressed, whereas AQP7 and AQP9 are downregulated in HCC, with AQP3 expression associated with aggressive tumor characteristics [129]. Research indicates that AQP3 levels are elevated in HCC tissues and this increase is inversely related to miR-124 expression [130]. The expression and localization of AQP9 are altered, and this is dependent on liver disease, with lower levels found in liver cancer tissues [131]. The loss of AQP8 and AQP9 contributes to resistance to apoptosis in HCC [132]. AQP5 is associated with tumor invasiveness, but its impact on prognosis is still unclear [133].

AQPs also play a critical role in other liver conditions and injuries. *Aqp9*^*−/−*^ knockout mouse models showed that silencing *Aqp9* decreased liver cell damage from excessive fat, thereby offering protection against subsequent inflammation, oxidative stress, apoptosis, and pyroptosis [134]. Leptin-deficient (ob/ob) mice, which serve as a model of nonalcoholic fatty liver disease (NAFLD, recently renamed as metabolic dysfunction-associated steatotic liver disease, MASLD [135–139]) exhibited diminished AQP9 expression and function following fasting, as well as elevated plasma glycerol levels compared to lean mice. This implies that AQP9 could be involved in the development of liver steatosis [93]. In a recent study with mice fed a high fat diet for 12 weeks, no evidence was found for AQP9 deficiency being associated with a reduced hepatic accumulation of TAGs or a diminished blood glucose level [96]. AQP9 involvement in hepatic steatosis may therefore depend on the mode and timing of pathogenesis. Reduced AQP9 expression has been found in liver biopsies from morbidly obese patients who are having bariatric surgery, with this being suggested as a potential protective mechanism against further liver fat accumulation [51].

Overexpression of AQP9 in a cell model of MASLD (recently induced by oleic acid in LO2 cells worsened steatosis, whereas silencing AQP9 reduced it [140]. Research using a HepG2 cell model consistently showed certain results. Furthermore, treatment with oleic acid resulted in increased phosphorylation of p38, and blocking p38 prevented upregulation of AQP9, indicating that AQP9 plays a role in oleic acid-induced hepatic steatosis in HepG2 cells through p38 signaling [141]. Data are also emerging that link AQP1 to arterial capillary growth in cirrhotic livers. In human cirrhotic and late-stage primary biliary cirrhosis (PBC) livers, AQP1 was primarily found in proliferating arterial capillaries, suggesting that AQP1 may trigger angiogenic responses. Increasing arterial blood flow into the sinusoids would raise sinusoidal microvascular resistance, exacerbating portal hypertension in patients with cirrhosis [142].

Experimental models of cholestasis such as extrahepatic obstructive cholestasis [143], estrogen-induced cholestasis [79], and sepsis-induced cholestasis [144] indicate that dysregulated AQP8 expression at the canalicular side of hepatocytes plays a key role in the development of cholestasis [43]. The downregulation of canalicular AQP8 and decreased canalicular osmotic water permeability indicate the role of AQP8 in cholestasis [79, 143]. Disrupted hepatocyte solute transporters and AQP8 function may compromise the relationship between osmotic gradients and canalicular water flow. These findings indicate that cholestasis can also arise from a simultaneous occurrence of decreased solute transport and disrupted water permeability [145]. The adenoviral transfer of the human AQP1 gene to a rat liver has been shown to improve bile flow in cases of estrogen-induced cholestasis, which may have potential therapeutic benefits for individuals with cholestatic diseases [146]. Matsumoto and colleagues discovered that a decrease in paracellular or transcellular canalicular water flow has no significant impact on BA excretion [80]. These findings support new theories about the beginning and development of cholestasis [147, 148]. For instance, AQP9 is a basolateral AQP known to facilitate the movement of water from the sinusoidal blood into the hepatocyte. It is worth noting that AQP9 expression was found to be reduced post-transcriptionally in a rodent model of extrahepatic cholestasis [88]. Human hepatic ischemia and hypoxia are accompanied by decreased bile flow, biliary sludge, and cholestasis, and this condition is associated with a reduction in the hepatocyte AQP8 protein at a post-translational level [149].

A rat model of autosomal recessive polycystic kidney disease showed hepatic cystogenesis linked to changes in the expression and subcellular placement of AQP1 (together with CFTR and AE2). It is likely that liver cysts grow due to increased fluid accumulation which is triggered by the overexpression and ectopic localization of AQP1, CFTR, and AE2 in cystic cholangiocytes [150]. In mice, disruption of the *Aqp11* gene resulted in the formation of intracellular vacuoles within periportal hepatocytes. This transgenic mouse developed a severe form of polycystic kidney disease (PKD) resulting in uremic death before weaning due to renal failure [60]. The lifespan of *Aqp11*^−/−^ mice was cut short due to kidney disease resulting from intracellular vacuolization of proximal tubular cells, potentially advancing the development of liver abnormalities. In *Aqp11* knockout mice, polycystic livers are anticipated due to the frequent occurrence of cysts in the biliary epithelia of PKD patients and mice [151]. Further investigation is necessary to determine whether the PKD resulting from the depletion of AQP11 in mice produces the same liver cysts as the autosomal recessive form of PKD, a well-documented form of PKD brought on by the homologous *Cpk* gene.

AQP3 was the only aquaglyceroporin identified in hepatic stellate cells, with HSC activation, such as in fibrosis, linked to decreased AQP3 expression [152]. A monoclonal antibody that inhibits AQP3 is being developed as a possible treatment for liver damage [153]. However, AQP3 was found to be the only aquaglyceroporin present in hepatic stellate cells (HSCs). HSC activation (e.g., in fibrosis) is associated with decreased AQP3 expression [152]. AQP3 is linked to the development and severity of extrahepatic cholangiocarcinoma [154].

### Gallbladder

Approximately 80% of gallstones consist of cholesterol [155], with the remainder being pigment stones that contain less than 30% cholesterol. Symptoms can develop within 5 years in approximately 10% of patients and within 20 years in approximately 20% of patients [156–158]. Transitioning from uncomplicated cholecystolithiasis to gallstone disease will raise the likelihood of recurring symptoms. Gallstone disease is also one of the most common and expensive digestive disorders in Western nations, affecting approximately 20% of adults [156, 159]. The incidence of cholesterol gallstones rises with age, occurs more frequently in women than men, and is linked to several risk factors [156, 160] such as insulin resistance, type 2 diabetes, the expansion of visceral adiposity resulting from being overweight and obese, and metabolic syndrome [161]. Gallstones can be largely considered a disease associated with metabolic dysfunction, as recently debated [158]. The development of cholesterol gallstones is the result of an interaction between several contributing factors and at least five primary defects. This includes the excessive production of cholesterol by the liver, resulting in bile that is highly saturated with cholesterol and prone to forming solid cholesterol deposits; genetic factors; and Lith genes, which increase a patient’s susceptibility to cholesterol gallstones through multiple mechanisms [162–165]; and rapid changes in biliary cholesterol that lead to the formation of early, solid, filamentous, and arc- or needle-like anhydrous cholesterol crystals and later, thermodynamically stable plate-like monohydrate crystals [166–170]. Additional factors that promote the formation of gallstones and gallbladder inflammation include bile stasis in the gallbladder, increased mucin secretion, and the accumulation of a mucin gel within the gallbladder's interior space. During cholesterol stone formation, gallbladder function can be disrupted, as supersaturated bile from the liver delivers large amounts of dissolved cholesterol to the gallbladder’s epithelial cells. In this context, cholesterol is converted into cholesteryl esters and stored in the mucosa and lamina propria. Excessive cholesterol in the smooth muscle plasmalemma causes the smooth muscle membrane to stiffen, thereby impairing the CCK-1 receptor signaling pathway. Chronic gallbladder inflammation and oxidative stress are also contributing factors [171, 172]. Factors within the intestines are responsible for the greater transport of cholesterol from the intestinal lumen to the liver and the intestine, where the resident colonic microbiota in the colon converts primary bile acids into secondary bile acids through biotransformation, resulting in elevated levels of biliary DCA. This BA is highly water-repelling, which leads to further excessive liver cholesterol production and cholesterol crystal formation.

Recent studies involving AQP8-depleted mice have found that gallstone formation occurred more quickly, and this accelerated process was alleviated by introducing AQP8 or AQP1 expression via an adenovirus in the liver [43]. The same work reported a small molecule, scutellarin, that increased the hepatocyte expression of AQP8 both in vitro and in vivo. Interestingly, in *Aqp8*^+/+^ wild-type mice, scutellarin significantly increased bile formation, reduced bile lipid concentrations, and prevented cholelithiasis compared to *Aqp8*^−/−^ knockout mouse littermates.

In the gallbladder of leptin-deficient obese Lep(ob) mice undergoing leptin replacement, reduced levels of *Aqp1* and *Aqp4* mRNA were detected [124]. In addition to displaying characteristic obesity, Lep(ob) mice exhibited increased gallbladder volumes and decreased gallbladder contractility, the latter indicating gallbladder stasis [126]. A study using immunohistochemistry investigated whether similar effects occur in humans with cholesterol gallstone disease, but found no significant link between the expression of AQP1 and AQP8 in the gallbladder lining and the presence or absence of gallstones [122]. Additional research is required to more effectively address the question, particularly in light of a recent study involving TH-deficient and cholestatic mice that demonstrated sex-specific expression and localization of hepatobiliary AQPs, where a lower cholesterol gallstone prevalence was observed in female C57BL/6J mice [173]. Lower expression of hepatobiliary AQPs was linked to reduced biliary water transport in male C57BL/6J mice and may have contributed to the sex-dependent cholesterol gallstone prevalence observed under TH deficiency conditions (Table 1).

**Table 1:** Role of AQPs in hepatobiliary diseases

Organ/disease	AQP member(s)	Expression/localization changes	Functional/pathophysiological role	Species/model	References
Liver—hepatocellular carcinoma (HCC)	AQP3	Overexpressed in tumor tissues; inversely correlated with miR-124	Promotes tumor growth, migration, and aggressiveness	Human	[129, 130]
	AQP8, AQP9	Underexpressed in HCC	Loss contributes to apoptosis resistance and tumor progression	Human	[131, 132]
	AQP5	Upregulated; localization linked to invasive phenotype	Associated with increased invasiveness; prognostic impact unclear	Human	[51, 93, 96, 133, 140]
Liver—metabolic dysfunction-associated steatotic liver disease (MASLD/NAFLD)	AQP9	Reduced expression in ob/ob and diet-induced obese mice; decreased in liver biopsies of obese humans	Regulates glycerol uptake and lipid synthesis; downregulation may protect from steatosis; overexpression exacerbates steatosis via p38 signaling	Mouse, Human, HepG2/LO2 cells	[51, 93, 96, 134, 140]. [141]
Liver—cirrhosis and portal hypertension	AQP1	Increased expression in proliferating arterial capillaries in cirrhotic and late-stage PBC livers	May promote angiogenesis, raise sinusoidal resistance, and worsen portal hypertension	Human	[142]
Cholestasis (extrahepatic, estrogen-, or sepsis-induced)	AQP8	Downregulated at the canalicular membrane	Reduced canalicular osmotic water permeability contributes to impaired bile secretion	Rat, Mouse	[43, 79, 143, 144]
	AQP9	Reduced post-transcriptionally	Decreased basolateral water flow from sinusoidal blood into hepatocytes	Rat	[88]
	AQP1	Adenoviral overexpression restores bile flow	Gene therapy improves bile flow in estrogen-induced cholestasis	Rat	[146]
	AQP8	Decreased protein in hypoxia/ischemia	Reduced bile flow and cholestasis due to post-translational downregulation	Human	[149]
Polycystic liver disease (in PKD models)	AQP1	Overexpressed and mislocalized in cystic cholangiocytes	Promotes cyst fluid accumulation and cystogenesis via AQP1–CFTR–AE2 axis	Rat	[150]
	AQP11	Loss-of-function mutation causes hepatocellular vacuolization and biliary cyst formation	Associated with PKD phenotype and early lethality from renal failure	Mouse	[60, 151]
Liver fibrosis and stellate cell activation	AQP3	Downregulated during hepatic stellate cell activation	May regulate HSC proliferation and activation; antibody inhibition under investigation as antifibrotic therapy	Human, mouse	[152, 153]
Extrahepatic cholangiocarcinoma	AQP3	Overexpressed	Associated with disease severity and poor prognosis	Human	[154]
Gallbladder—cholesterol gallstone disease	AQP8, AQP1	AQP8 deficiency accelerates gallstone formation; adenoviral AQP8 or AQP1 rescues phenotype	AQPs facilitate bile fluidity; scutellarin upregulates AQP8 and prevents cholelithiasis	Mouse	[43]
	AQP1, AQP4	Reduced mRNA levels in leptin-deficient (ob/ob) mice after leptin treatment	Associated with impaired gallbladder contractility and stasis	Mouse	[124, 126]
	AQP1, AQP8	No significant difference between gallstone and non-gallstone patients	Unclear involvement in human gallstone formation	Human	[122]
	Various AQPs	Sex-specific differences in AQP expression/localization	Reduced AQP expression in males linked to lower biliary water transport and sex-dependent gallstone prevalence	Mouse	[173]
Gallbladder cancer (GBC)	AQP5 (and others)	Overexpressed in GBC tissues	Potential role in tumor cell proliferation, invasion, and metastasis; therapeutic target under investigation	Human	[174, 175]

Attention is increasingly being focused on the role of AQPs, particularly AQP5, in gallbladder cancer (GBC) due to their potential to promote cancer cell growth, invasion, and metastasis. Despite their established importance in other types of cancer, there is limited research available on the particular mechanisms and clinical significance of AQPs in gallbladder cancer. Initial research implies that AQP5 could impact tumor behavior and patient outcomes; however, additional studies are required to elucidate the full scope of AQPs and their potential as therapeutic targets in GBC [174, 175].

## New modulators in the pharmacological targeting of AQPs

Development of drugs targeting AQPs has been hindered, which might be attributed to several assumptions, including the notion that AQP pores are inherently resistant to drug targeting, and this notion has been reinforced by difficulties in replicating current experimental procedures [176]. Drug development targeting AQP has become a rapidly expanding field in recent years, driven in part by the promising use of both natural and synthetic compounds that can selectively block or modulate the AQPs’ channel, expression, or regulatory mechanisms [177–182]. Developing new drugs targeting AQPs to treat conditions characterized by an imbalance of water and solutes in the body is crucial due to the lack of existing pharmacological treatments [183, 184].

The compiled data highlight the diversity of currently available AQP inhibitors with respect to chemical class, potency, and isoform selectivity. Both classical metal-based inhibitors (e.g., mercury(II) chloride, silver nitrate, copper(II) sulfate, and nickel(II) chloride) and organic small molecules (e.g., RG100204, TGN-020, acetazolamide, phloretin, and bumetanide) demonstrate inhibitory activity across multiple AQP isoforms (Table 2). Notably, several compounds exhibit isoform preference, such as TGN-020 toward AQP4 [185] and RG100204 toward AQP9 [86, 186], whereas others, including phloretin [187, 188] and certain metals [22, 189–192], show broader activity profiles. Reported IC₅₀ values in the submicromolar range for selected compounds (e.g., RG100204 in CHO cells) suggest relatively high potency, although inhibitory effects are strongly influenced by the experimental model and assay conditions [193]. The variability in concentrations required for inhibition across different systems further underscores the complexity of AQP pharmacology and the challenges associated with achieving isoform selectivity and translational relevance.

**Table 2:**
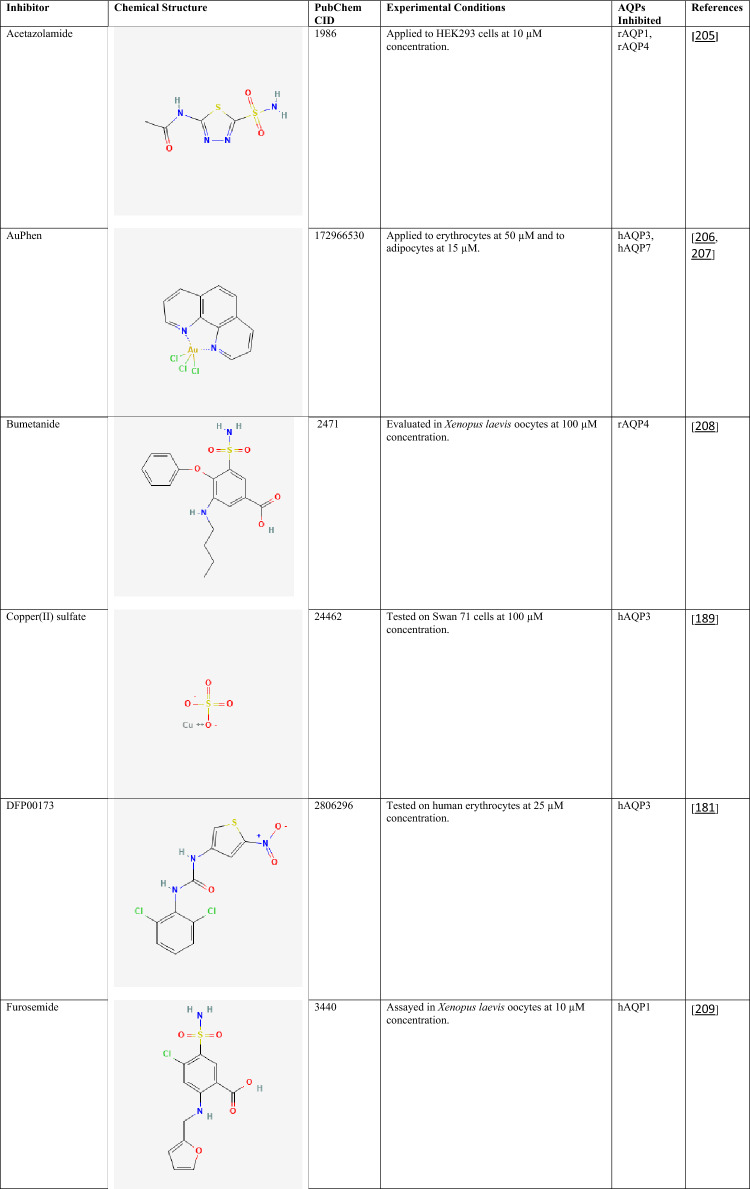

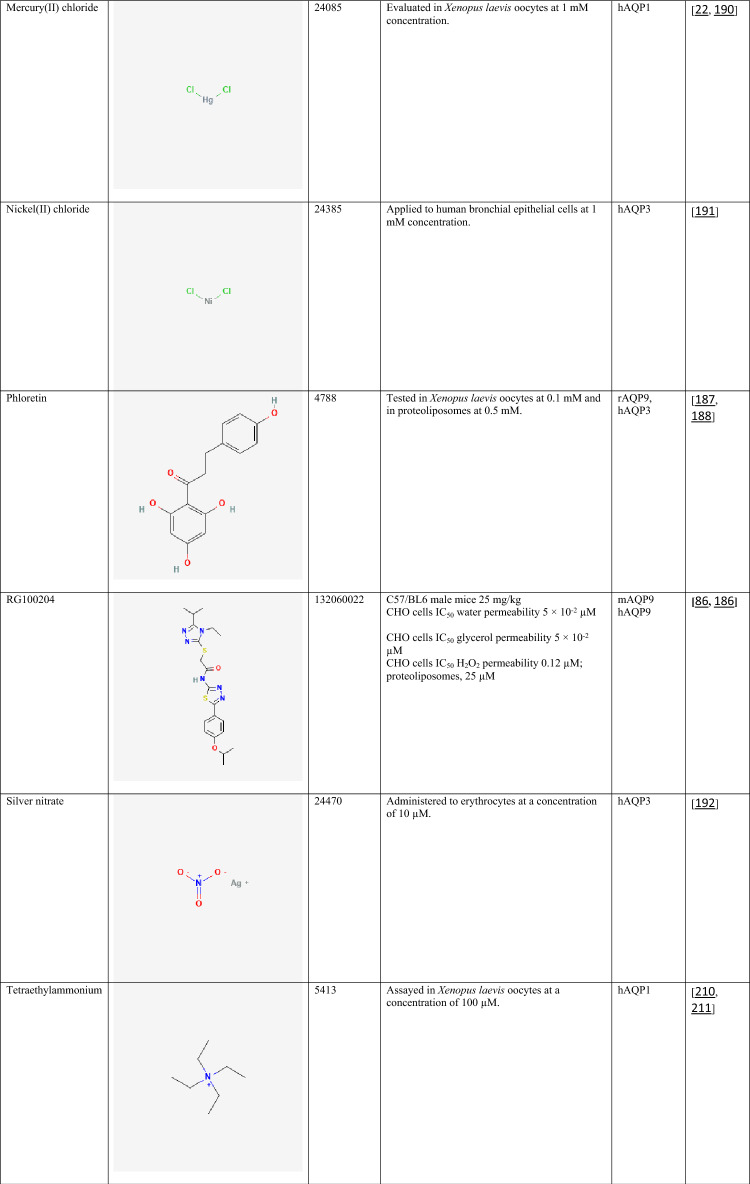

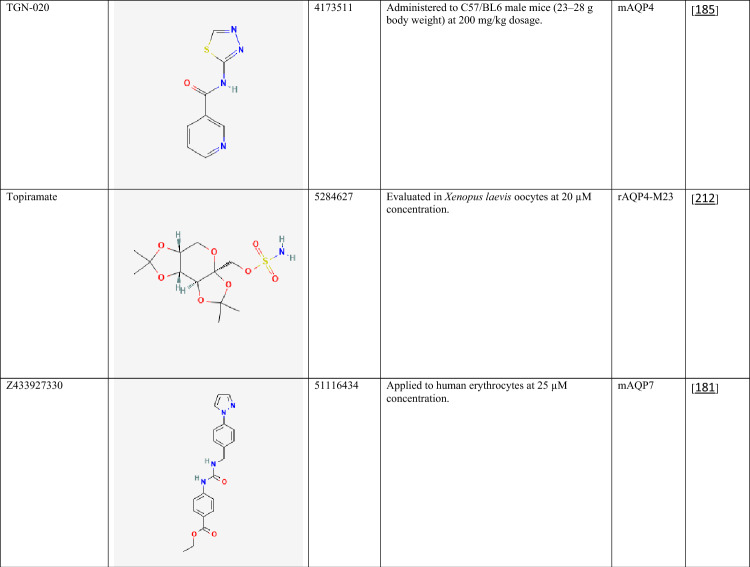
Chemical structures and pharmacological profiles of selected AQP inhibitors

All chemical structures were obtained from PubChem (https://pubchem.ncbi.nlm.nih.gov)

AQP, aquaporin; CHO, Chinese hamster ovary; CID, compound Identifier; HEK293, human embryonic kidney 293 cells; H_2_O_2_, hydrogen peroxide; hAQP, human aquaporin; IC₅₀, half maximal inhibitory concentration; mAQP, mouse aquaporin; rAQP, rat aquaporin

Studies conducted prior to clinical trials, encompassing cellular and animal research, demonstrated that various artificial and natural substances can influence AQPs in diverse disease models [100, 179, 194, 195]. Preventive and therapeutic strategies of value could be derived from AQP modulators that may target the expression of AQP1 and AQP8 in the liver, in cholangiocytes, and in the gallbladder epithelium. The subsequent modulation of bile concentration is especially beneficial in clinical conditions such as cholestasis [43, 145, 146, 196] (e.g., cholestatic liver diseases, biliary cirrhosis, cholangitis) and in individuals at high risk of developing gallstones. Targeting AQP1 through gene therapy represents a promising strategy for enhancing fluid flow in liver and gallbladder. The rationale stems from evidence that AQP1 facilitates transcellular water movement, and its overexpression in salivary glands has been shown to significantly increase water flow into the ductal lumen. In studies addressing radiation-induced salivary gland hypofunction, adenovirus-mediated AQP1 gene transfer effectively improved saliva secretion by enhancing water permeability across ductal epithelial cells, which remain largely intact after irradiation [176]. Both animal models (rats [197] and miniature pigs [198]) and clinical trials in humans [199, 200] demonstrated that AQP1 expression can partially restore fluid secretion, confirming its physiological role in driving osmotic water transport. Thus, the success of AQP1 gene transfer in salivary glands provides proof of concept that modulating AQP1 expression could serve as a viable therapeutic strategy to enhance bile flow and fluid secretion in liver and gallbladder diseases associated with reduced water permeability.

**Fig. 3:**
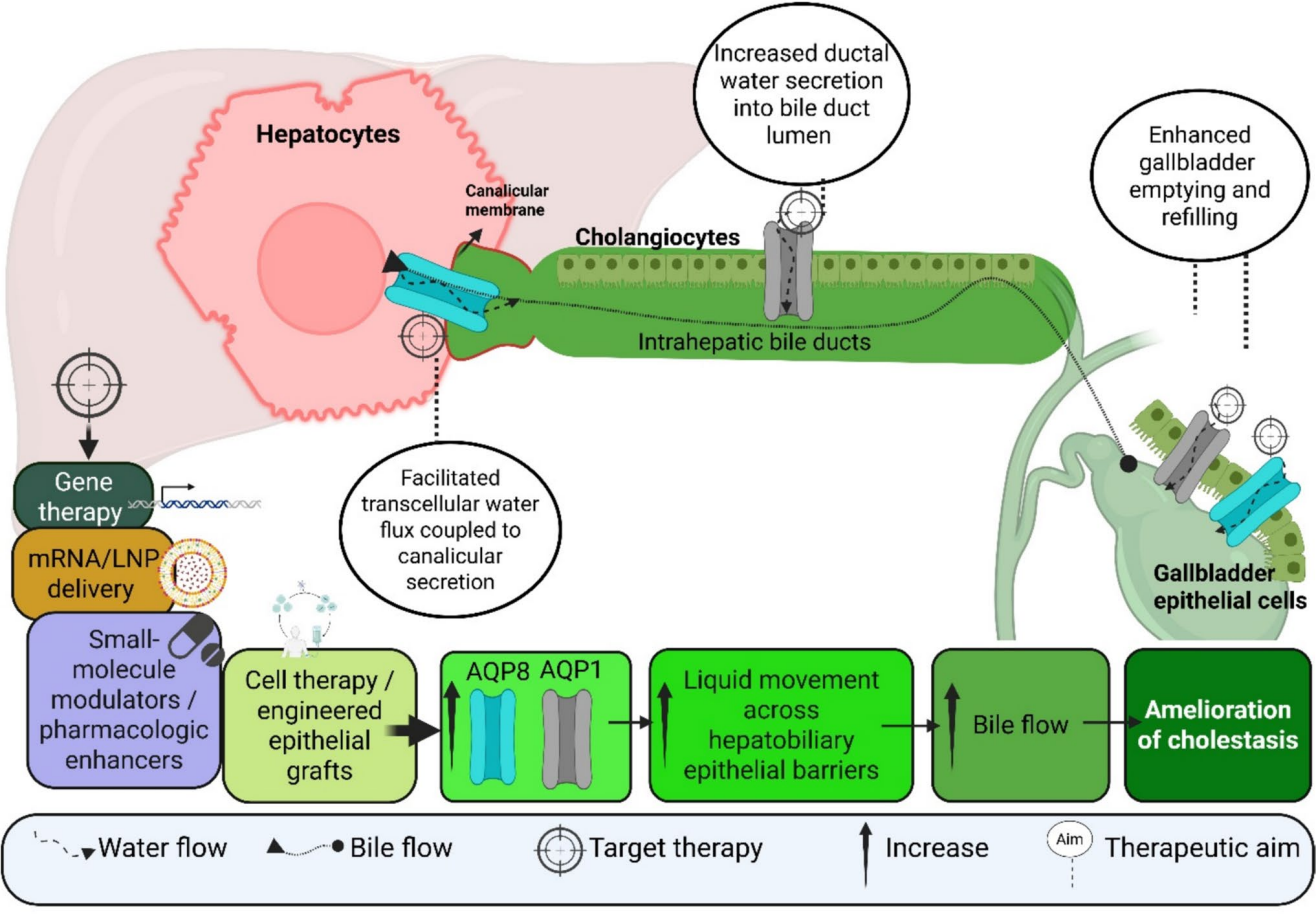
The diagram illustrates the proposed therapeutic targeting of aquaporins AQP1 and AQP8 in hepatocytes, cholangiocytes, and gallbladder epithelial cells to enhance water and bile flow across the hepatobiliary tract. Canalicular membrane AQP8 in hepatocytes facilitates transcellular water flux coupled to canalicular secretion, while AQP1, expressed in cholangiocytes and gallbladder epithelial cells, increases ductal water secretion and promotes gallbladder bile filling and emptying. Potential therapeutic strategies include gene therapy, mRNA/LNP delivery, small-molecule modulators, and cell or engineered epithelial grafts designed to upregulate or deliver AQP1 and AQP8. Together, enhanced AQP expression promotes liquid movement across hepatobiliary epithelial barriers, resulting in increased bile flow and ultimately alleviation of cholestasis. Created in https://BioRender.com

Figure 3 illustrates that targeting AQP1 and AQP8 may help to enhance bile flow, potentially reducing symptoms and local inflammation.

Targeting AQP1 and AQP8 in diseases affecting bile secretion and flow may be an effective approach to enhance water homeostasis and manage bile volume.

The activation of the nuclear receptor FXR may also have additional benefits as a result of AQP8 overexpression. Animal studies indicate this pathway should influence fat production in the liver, leading to reduced fat accumulation in subjects with steatosis [75].

Preliminary findings suggest that high levels of AQP1 and AQP5 expression are beneficial for individuals with biliary tract cancer, particularly in terms of longer survival rates, smaller tumor sizes, and less invasive tumors [175]. Nevertheless, we need to be cautious when interpreting results in the background of additional data implying a connection between AQP overexpression and cancer-promoting effects in various types of cancer, including skin [201], breast [202], gastric [203], and colon cancer [204]. In contrast, knocking down AQPs can suppress tumorigenesis in vivo and inhibit the proliferative, migratory, and self-renewal capability of cancer cells [203, 204].

## Conclusions and research trends

The majority of AQPs is expressed in the digestive system and has significant implications for the physiopathology of the gastrointestinal tract. In the hepatobiliary system, aquaporins play a vital role in maintaining the precise balance of bile composition and flow, and bile water secretion and reabsorption, as well as plasma glycerol uptake by the hepatocyte and its conversion to glucose during starvation. Evidence is accumulating that AQPs may also be involved in the secretion of bile into canaliculi and ducts, gluconeogenesis, and microbial infection, and they may have additional novel roles impacting liver function. Research findings from basic and translational studies have shown that the dysregulation of AQP expression or function is linked to several hepatobiliary disorders, such as cholestatic liver diseases and cancer. Recent progress in our understanding of AQP biology and physiology has enabled the identification of potential therapeutic targets and possible side effects.

Recent studies have suggested AQP-targeted therapies, including the creation of AQP inhibitors or modulators, which are clinically relevant; however, clinical trials are currently non-existent and numerous obstacles remain. Indeed, researchers are investigating the role of small molecule modulators to revive or control AQP function, thus reducing abnormal bile secretion patterns seen in diseases affecting the liver’s bile ducts. The use of modulators for AQP1 and AQP8 could potentially help to improve bile flow and offer novel therapeutic strategies by addressing the root causes of bile secretion-related disorders. Additional basic research and clinical trials are still required to convert these promising therapeutic options into effective treatments.

## Supplementary Information

Below is the link to the electronic supplementary material.

Supplementary Fig. 1 Flow diagram of the study selection process. A total of 1,294 records were identified through database searching, with 513 duplicates removed. Titles and abstracts of 781 records were screened, excluding 455 studies due to non-hepatobiliary focus, non-AQP studies, or non-relevant scope. Full-text articles of 326 records were assessed for eligibility, of which 158 were excluded for being non-hepatobiliary, descriptive without functional or mechanistic data, or redundant/superseded. This resulted in 168 records included, with an additional 47 key articles and reviews on AQP inhibitors manually added, yielding 212 final records included in the review. (JPG 100 KB)

Supplementary Fig. 2 Aquaporin-mediated water transport in bile formation and modification. (A) In hepatocytes, aquaporins (AQPs) localized at the canalicular and basolateral membranes facilitate osmotic water movement during bile formation. Active secretion of bile salts and electrolytes into the bile canaliculus generates osmotic gradients that drive transcellular water flow. Hormonal stimulation (e.g., glucagon) activates the cAMP–PKA pathway, promoting exocytotic insertion of AQPs from subapical vesicles into the canalicular membrane, thereby increasing membrane water permeability and supporting bile salt–dependent bile flow. **(B)** In cholangiocytes, secretin-induced cAMP signaling stimulates CFTR-mediated Cl⁻ secretion and AE2-dependent Cl⁻/HCO₃⁻ exchange at the apical membrane, producing bicarbonate-rich ductal bile. The resulting osmotic gradient drives water secretion through apical and basolateral AQPs, contributing to bile salt–independent bile flow. AQP trafficking to the apical membrane is regulated by microtubule-dependent vesicular transport. **(C)** In gallbladder epithelial cells, AQPs expressed at both apical and basolateral membranes mediate rapid bidirectional water movement. Osmotically driven water reabsorption from the lumen to the serosal side enables bile concentration during storage. Together, these coordinated and cell-specific mechanisms highlight the essential role of AQPs in regulating bile volume, composition, and flow throughout the hepatobiliary system. (JPG 89 KB)
