# Supplementary figures and images for: Aquaporin membrane channels in the hepatobiliary tract: a model of complexity and clinical implications in health and disease
Source: Intern Emerg Med. 2026 Apr 2;21(3):807–29. doi: 10.1007/s11739-026-04332-y (PMC13144280; doi:10.1007/s11739-026-04332-y)

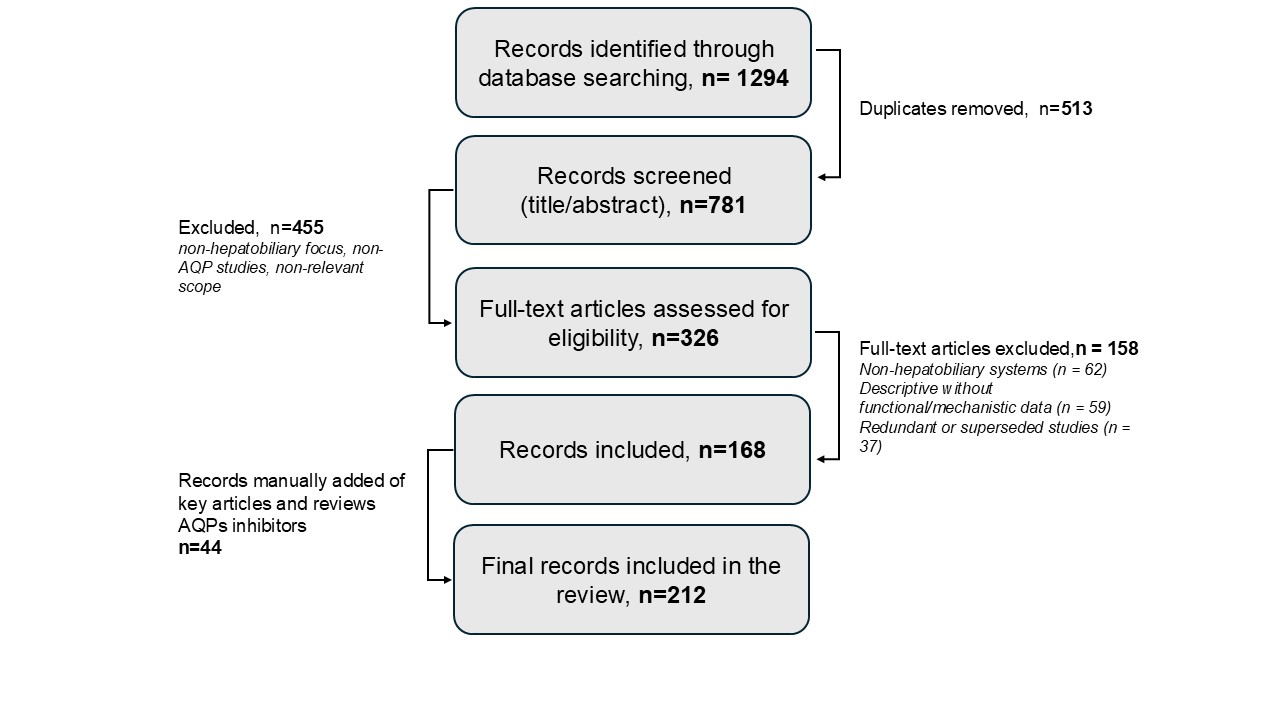

Supplement: Supplementary file 1 — Supplementary Fig. 1 Flow diagram of the study selection process. A total of 1,294 records were identified through database searching, with 513 duplicates removed. Titles and abstracts of 781 records were screened, excluding 455 studies due to non-hepatobiliary focus, non-AQP studies, or non-relevant scope. Full-text articles of 326 records were assessed for eligibility, of which 158 were excluded for being non-hepatobiliary, descriptive without functional or mechanistic data, or redundant/superseded. This resulted in 168 records included, with an additional 47 key articles and reviews on AQP inhibitors manually added, yielding 212 final records included in the review. (JPG 100 KB) [file 11739_2026_4332_MOESM1_ESM.jpg]

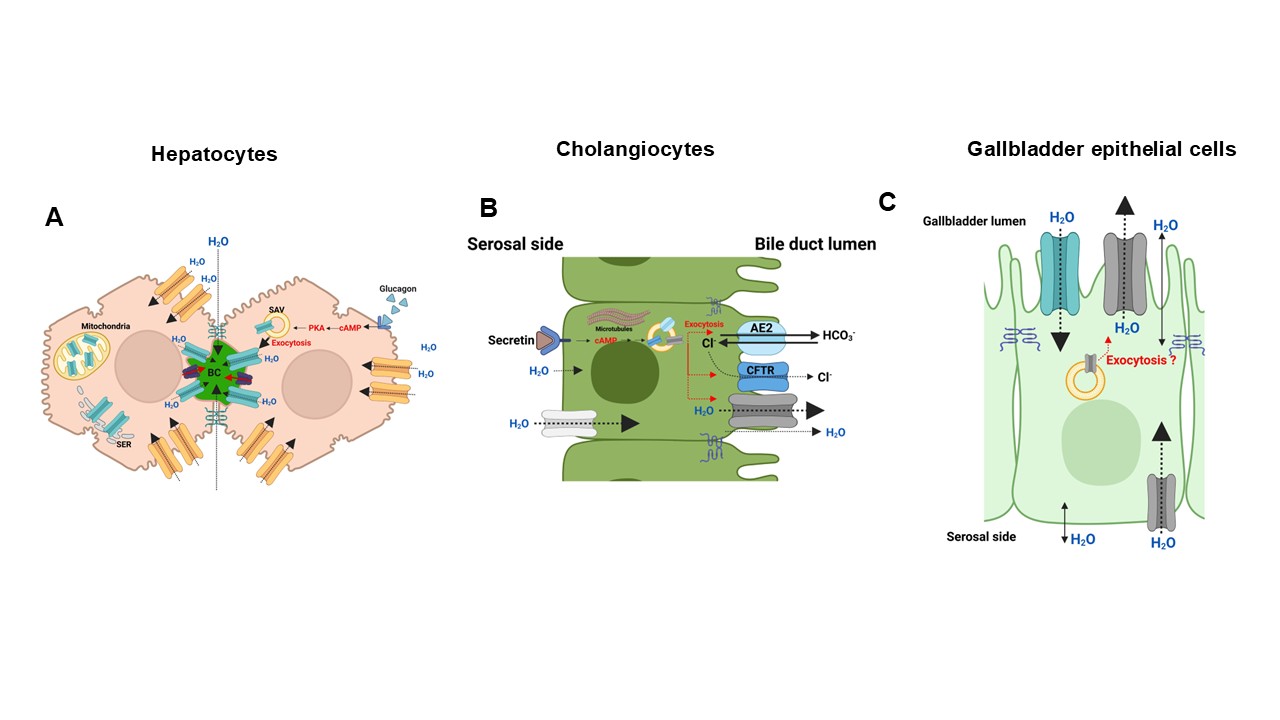

Supplement: Supplementary file 2 — Supplementary Fig. 2 Aquaporin-mediated water transport in bile formation and modification. (A) In hepatocytes, aquaporins (AQPs) localized at the canalicular and basolateral membranes facilitate osmotic water movement during bile formation. Active secretion of bile salts and electrolytes into the bile canaliculus generates osmotic gradients that drive transcellular water flow. Hormonal stimulation (e.g., glucagon) activates the cAMP–PKA pathway, promoting exocytotic insertion of AQPs from subapical vesicles into the canalicular membrane, thereby increasing membrane water permeability and supporting bile salt–dependent bile flow. (B) In cholangiocytes, secretin-induced cAMP signaling stimulates CFTR-mediated Cl⁻ secretion and AE2-dependent Cl⁻/HCO₃⁻ exchange at the apical membrane, producing bicarbonate-rich ductal bile. The resulting osmotic gradient drives water secretion through apical and basolateral AQPs, contributing to bile salt–independent bile flow. AQP trafficking to the apical membrane is regulated by microtubule-dependent vesicular transport. (C) In gallbladder epithelial cells, AQPs expressed at both apical and basolateral membranes mediate rapid bidirectional water movement. Osmotically driven water reabsorption from the lumen to the serosal side enables bile concentration during storage. Together, these coordinated and cell-specific mechanisms highlight the essential role of AQPs in regulating bile volume, composition, and flow throughout the hepatobiliary system. (JPG 89 KB) [file 11739_2026_4332_MOESM2_ESM.jpg]
